# Supplementary material for: End-product inhibition of the LRRK2-counteracting PPM1H phosphatase
Source: bioRxiv. 2025 May 17:2025.05.16.654599. Preprint. [Version 1] doi: 10.1101/2025.05.16.654599 (PMC12132568; doi:10.1101/2025.05.16.654599)
Supplement: 1 [file NIHPP2025.05.16.654599V1-supplement-1.pdf]

bioRxiv preprint doi: https://doi.org/10.1101/2025.05.16.654599; this version posted May 17, 2025. The copyright holder for this preprint (which was not certified by peer review) is the author/funder, who has granted bioRxiv a license to display the preprint in perpetuity. It is made available under aCC-BY 4.0 International license.

Figure S1. Proteins used in this study. A. Alphafold model of mNeon PPM1H superimposed on wild type PPM1H full length protein. B-D., SDS-PAGE of the gel filtration step of protein purification for (B) mNeon-PPM1H, (C) mNeon L66R PPM1H and (D) mNeon Δ37 PPM1H. Numbers at right indicate molecular weight marker mobility shown in kDa. Also shown are the preparations before and after cleavage to remove the His-Sumo tags.

Figure S2. PhosTag gel electrophoresis to monitor MST phosphorylation of Rab8A with ATP (A, left half) or ATPγS (A, right half) as indicated. ATP Reactions were carried out for either 2h or overnight as indicated; ATPγS reactions were carried out overnight; two samples were further treated with PPM1H for 15 minutes at 30°C to check for thiophosphate stability to phosphatase action. B, Quantitation of the gel in A. PhosTag gels were purchased from Fuji Film Wako pure Chemical Corporation, Osaka (195-17991); samples were loaded in sample buffer containing LDS instead of SDS and 1mM $ZnCl_2$, at 120V for 2hr.

bioRxiv preprint doi: https://doi.org/10.1101/2025.05.16.654599; this version posted May 17, 2025. The copyright holder for this preprint (which was not certified by peer review) is the author/funder, who has granted bioRxiv a license to display the preprint in perpetuity. It is made available under aCC-BY 4.0 International license.

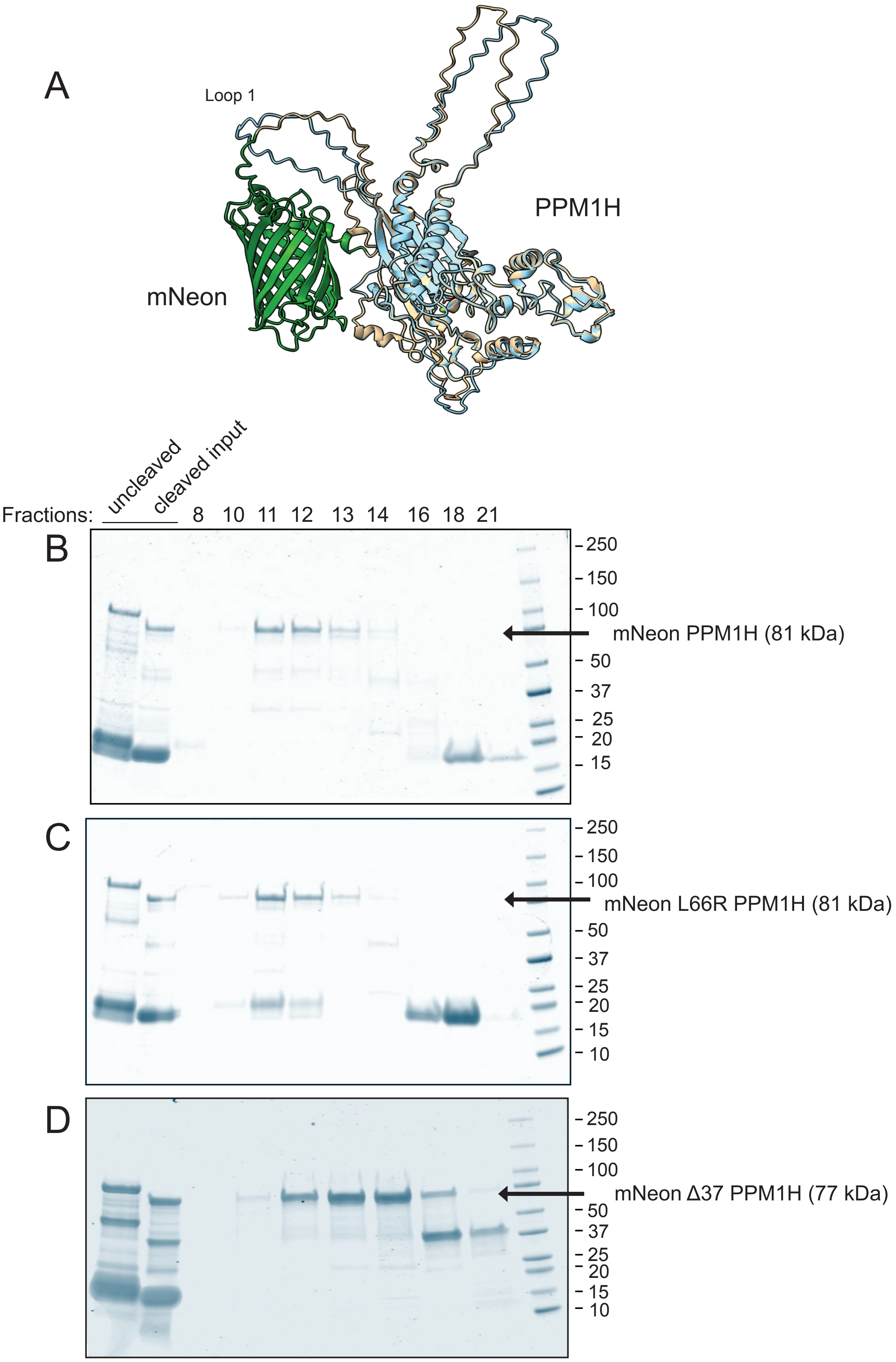

Adhikari et al., Figure S1

bioRxiv preprint doi: https://doi.org/10.1101/2025.05.16.654599; this version posted May 17, 2025. The copyright holder for this preprint (which was not certified by peer review) is the author/funder, who has granted bioRxiv a license to display the preprint in perpetuity. It is made available under aCC-BY 4.0 International license.

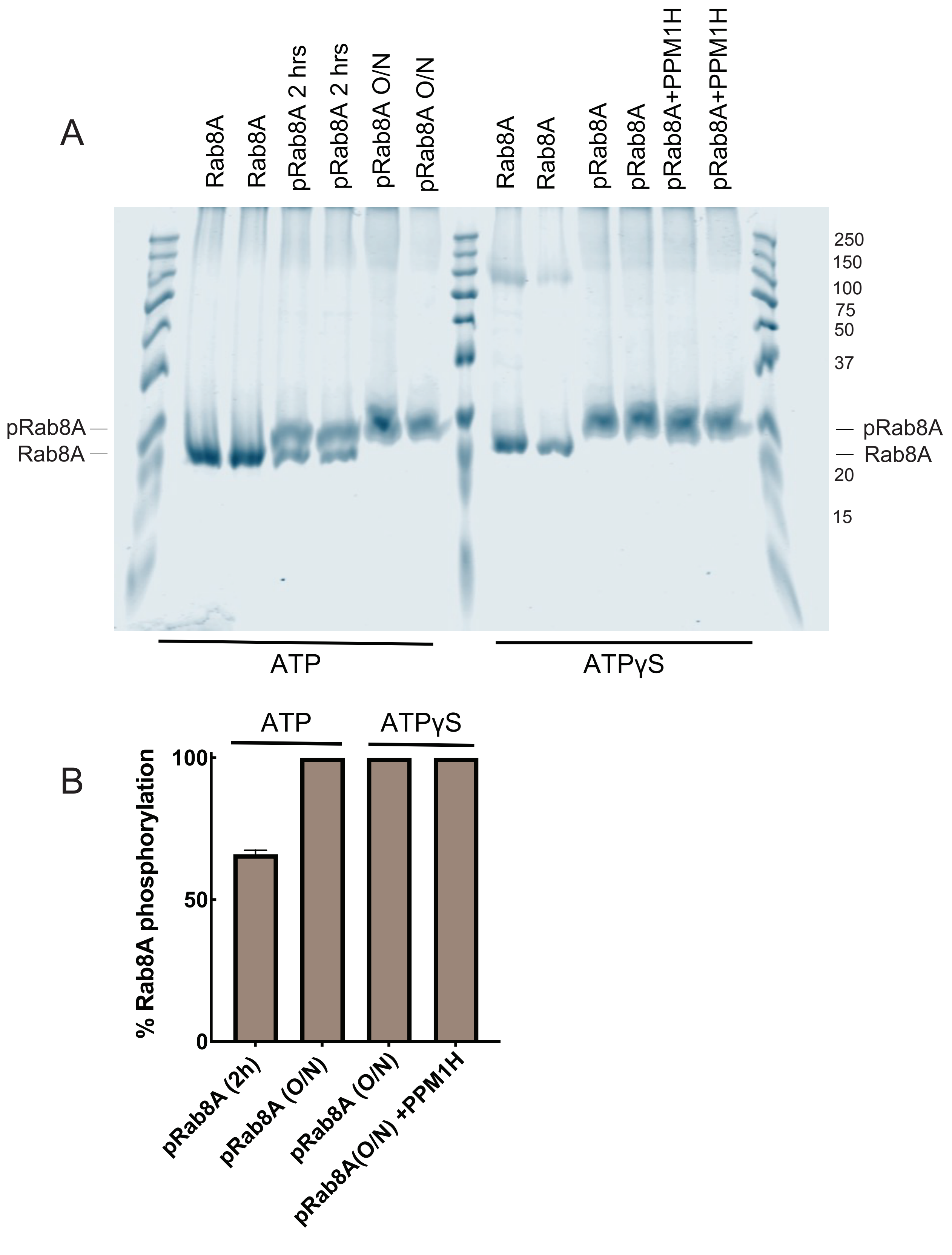

Adhikari et al., Figure S2
